# Anatomic variations in retaining ligaments during minimally invasive resection of subcutaneous lipomas

**DOI:** 10.3205/iprs000172

**Published:** 2023-07-07

**Authors:** Akio Sakamoto, Takashi Noguchi, Shuichi Matsuda

**Affiliations:** 1 Department of Orthopaedic Surgery, Graduate School of Medicine, Kyoto University, Japan

**Keywords:** lipoma, operative time, technique, minimally invasive, magnetic resonance imaging

## Abstract

**Objective:**

Lipomas are common, benign tumors usually located in the subcutaneous tissue. The “one-inch method” is a minimally invasive technique for resecting large subcutaneous lipomas through a one-inch incision after blunt dissection of the lipoma from its peripheral retaining ligaments. The limitations of this method are currently unclear.

**Materials and methods:**

We assessed twenty-five patients with large lipomas, defined as a tumor diameter greater than 5 cm. The location of the lipoma was at the shoulder in fifteen patients, the extremity in six patients, and the torso in four patients.

**Results:**

The mean operative time for all lesions was 28.3 minutes, with a mean time of 25.9 minutes for lipomas at the shoulder, 21.8 minutes for the extremities, and 47.0 minutes for the torso. We classified patients into three groups according to operative time: the short group (10–29 min), middle group (30–49 min), and long group (50–70 min). For lipomas of the shoulder, there were eleven patients (73%) in the short group, three patients (20%) in the middle group, and one patient (7%) in the long group. For lipomas of the extremity, the groups contained five patients (83%), one patient (17%), and no patients (0%), respectively. For lipomas of the torso, the groups contained one patient (25%), no patients (0%), and three patients (75%), respectively.

**Conclusions:**

Lipomas of the torso require a longer operative time than those of the shoulder or extremity; this difference could be due to the number of retaining ligaments present, which is reportedly higher in the back than in the anterior or side body. Lipomas of the back are less amenable to the one-inch method, and posterior shoulder lipomas may take more time than those at other parts of the shoulder or at the extremities.

## Zusammenfassung

**Zielsetzung:**

Lipome sind häufig auftretende, gutartige Tumore, die sich in der Regel im subkutanen Gewebe befinden. Bei der „Ein-Zoll-Methode“ handelt es sich um eine minimal-invasive Technik zur Resektion großer subkutaner Lipome durch eine Ein-Zoll-Inzision nach stumpfer Dissektion des Lipoms von seinen peripheren Bändern. Die Grenzen dieser Methode sind derzeit unklar.

**Material und Methoden:**

Wir untersuchten 25 Patienten mit großen Lipomen, definiert als Tumore mit einem Durchmesser von mehr als 5 cm. Bei 15 Patienten befand sich das Lipom an der Schulter, bei sechs Patienten an den Extremitäten und bei vier Patienten am Rumpf.

**Ergebnisse:**

Die mittlere Operationszeit für alle Läsionen betrug 28,3 Minuten mit einer mittleren Zeit von 25,9 Minuten für Lipome an der Schulter, 21,8 Minuten für die Extremitäten und 47,0 Minuten für den Rumpf. Die Patienten wurden nach der Operationszeit in drei Gruppen eingeteilt: Gruppe 1 (kurze Operatiosnzeit: 10–29 Minuten), Gruppe 2 (mittlere Operationszeit: 30–49 Minuten), Gruppe 3 (lange Operationszeit: 50–70 Minuten). Bei den Lipomen der Schulter waren elf Patienten (73%) in Gruppe 1, drei Patienten (20%) in Gruppe 2 und ein Patient (7%) in Gruppe 3. Bei den Lipomen an den Extremitäten entfielen auf die Gruppen fünf Patienten (83%), ein Patient (17%) und kein Patient (0%). Bei den Lipomen am Rumpf entfielen auf die Gruppen ein Patient (25%), kein Patient (0%) bzw. drei Patienten (75%).

**Schlussfolgerungen:**

Lipome am Rumpf erfordern eine längere Operationszeit als solche an der Schulter oder den Extremitäten; dieser Unterschied könnte auf die Anzahl der vorhandenen Bänder zurückzuführen sein, die am Rücken bekanntermaßen höher ist als am vorderen oder seitlichen Körper. Lipome am Rücken lassen sich weniger gut mit der Ein-Zoll-Methode entfernen und Lipome an der hinteren Schulter können mehr Zeit in Anspruch nehmen als Lipome an anderen Teilen der Schulter oder an den Extremitäten.

## Introduction

Lipomas are benign, slow-growing tumors that are usually located in the subcutaneous tissue. The histology is characterized by proliferation of adipose tissue, and the diagnosis can be made using magnetic resonance imaging (MRI). Lipomas are characterized by high signal intensity on T1- and T2-weighted images, which is similar to the appearance of normal subcutaneous adipose tissue.

Surgical excision is the mainstay of treatment for lipomas [1], but the decision to resect depends of the degree of bother to the patient, including cosmetic annoyance. Resection frequently requires incisions equal to the diameter of the tumor. When lipomas are large, this leads not only to a cosmetically undesirable incision, but also a risk for chronic postoperative pain at the incision site and hypoesthesia around the incision. Small incisions for large lipomas would be cosmetically preferable [2] and could possibly decrease postoperative pain and sensory disturbances. 

The “squeeze technique” involves resecting lipomas through a small incision overlying the tumor. This technique is well described for lipomas of the forearm or leg, but it is frequently unsuccessful for large lipomas, particularly those located at the shoulder [3], [4]. Liposuction has also been reported as a potential option for minimally invasive treatment. However, the long-term results of liposuction are disappointing in terms of completeness of resection and frequency of side effects, especially when the lipoma contains fibrous tissue [2], [5]. This tissue is actually subcutaneous perpendicular fibrous septae – or retaining ligaments – that are normal membranous structures located between the fascia and the skin [5]. These retaining ligaments intrude into the lipoma from its periphery and demonstrate low signal intensity on T1- and T2-weighted MRI [5].

As with liposuction, when the squeeze technique fails, it is because the lipoma is trapped by these peripheral retaining ligaments. The “one-inch method” was invented to overcome this difficulty [5]. This method uses a one-inch incision that allows the lipoma to be detached from its peripheral retaining ligaments using blunt digital dissection. The released tumor is then removed through the incision using either the squeeze technique or piece-by-piece removal [5]. The one-inch method is able to achieve success, even for large lipomas of the shoulder that are less amenable to the squeeze technique alone [5]. The small incision resolves the cosmetic issues related to longer incisions and is more likely to preserve sensation. The retaining ligaments might contain branches of cutaneous nerves, and cutting these nerves might contribute to hypoesthesia. In a previous report describing retaining ligaments in the face, these ligaments were identified as serving a sentinel role for peripheral nerve branches [6], [7]. 

While the one-inch method is easy in theory, surgeons sometimes encounter difficulty with detaching and removing the tumor. The process of blunt dissection may cause pain and annoyance to the patient during the procedure. Therefore, general anesthesia is typically required to use the one-inch method for large lipomas. However, local anesthesia might be possible if surgeons know in advance that the procedure is likely to be easy with a short operative time. 

The purpose of this study is to identify factors that are related to difficulties performing the one-inch method and thereby identify when this method might not be indicated, and when it is indicated. When surgery for lipoma is expected to be easier, the procedure could be performed under local anesthesia. With the one-inch method, the operative time depends on the difficulty of tumor removal – in particular, the difficulty of detaching the lesion from the retaining ligaments. 

We conducted a retrospective analysis of a series of patients who underwent removal of subcutaneous large lipomas using the one-inch method. We assessed the difficulty of the operation, using the operative time as a proxy for difficulty. 

## Materials and methods

We conducted a retrospective review to identify patients with lipomas that had a diameter equal to or greater than 5 cm and that were resected using the one-inch method. The radiographic diagnosis of lipoma was made by MRI, with lipomas demonstrating high signal intensity on T1- and T2-weighted images. High signal intensity was not present on T2-weighted images when fat suppression was used. Lipoma removal using the one-inch method was performed by a single surgeon (AS), who was trained in the procedure by the inventor of the method [5]. The diagnosis of lipoma was confirmed on pathologic examination of the resected material.

**Figure 1:**
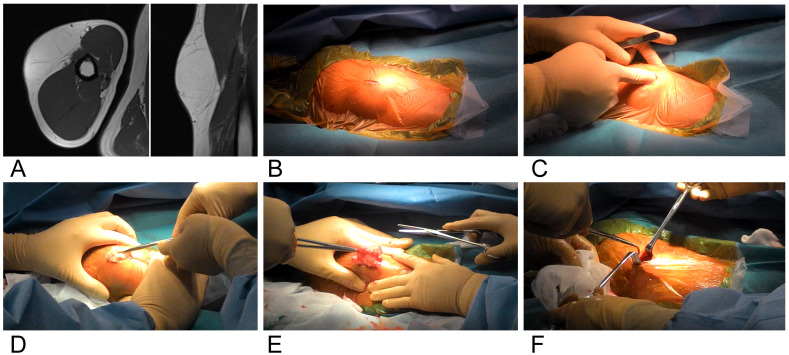
A 33-year-old male with a lipoma of the upper arm. The MRI shows a lesion with a high-intensity signal on T1-weighted images, and the signal pattern is similar to subcutaneous adipose tissue. A: Retaining ligaments are demonstrated as lines with low-intensity signal. B: In the operation, a one-inch incision is made at the center of the lipoma. C: Detachment of the lipoma from the retaining ligaments is performed bluntly with a finger. D and E: The released lipoma is extracted in a piecemeal fashion or by using a “squeeze technique”. F: Compote resection can be confirmed visually through the incision given the skin laxity. Residual lipomas can be easily removed with forceps.

The technique used for the one-inch method for removal of subcutaneous lipomas has been reported previously [5]. The patient is placed under general anesthesia, and a one-inch incision is made over the lipoma. Blunt dissection, using a finger, is used to detach the lipoma from any retaining ligaments. The released lipoma is then extracted, in a piecemeal fashion or using the squeeze technique, through the incision. Detachment and extraction are repeated until resection is complete. Complete resection is easily confirmed by visual inspection through the incision given the laxity of the skin once the bulk of the lipoma is removed. The subcutaneous tissue is approximated, and the skin incision is sutured using 3-0 or 4-0 nonabsorbable suture (Figure 1 (Fig. 1)).

We assessed the operative time for each patient and analyzed the time required according to patient demographic data and tumor location. Patients were then divided into three groups based on operative time: the short group (10–29 min), the middle group (30–49 min), and the long group (50–70 min). The grouped operative time was also analyzed for any associations with patient demographic data.

## Statistical analysis

The operative times were compared with clinical features using the Mann-Whitney U test for quantitative data and the chi-squared test for qualitative data. A p value of less than 0.05 was considered to indicate statistical significance.

## Results

A total of twenty-five patients were analyzed: Eighteen women and seven men with a mean age of 50.8 years (range: 26–72 years). The mean lipoma diameter was 6.8 cm (range, 5–13 cm). The lipoma location was at the shoulder in fifteen patients (60%), the extremity in six patients (24%), and the torso in four patients (16%). Of the six extremities involved, four were the upper arm and two were the thigh. Lesions of the torso involved the chest wall in two cases and the back in two cases. The fifteen shoulder lesions were located at the anterior shoulder in two, the lateral aspect in four, the cranial aspect in two, and the posterior aspect in seven. 

The mean operative time for all lesions was 28.3 minutes (range: 10–62 min). All data are shown in Table 1 (Tab. 1). The operative time was not correlated with patient age, sex, or tumor size. The mean operative time for lesions of the shoulder was 25.9 minutes (range: 10–59 min). The mean operative time for lesions of the extremity was 21.8 minutes (range: 15–32 min). The mean operative time for lesions of the torso was 47.0 minutes (range: 19–62 min). Although the mean time for lesions of the torso was longer than for those of the shoulder (p=0.07) and those of the extremities (p=0.08), the difference was not statistically significant. 

**Figure 2:**
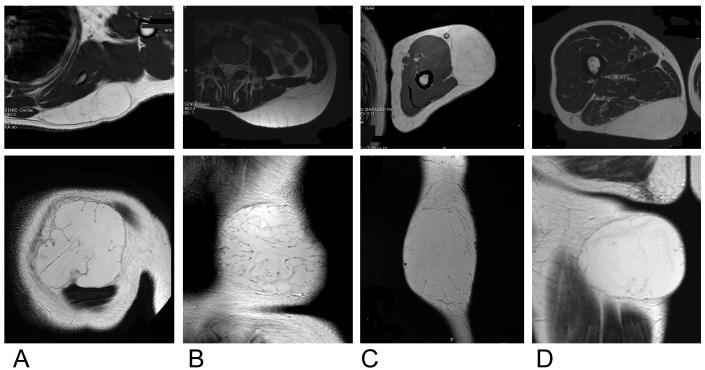
A: A 37-year-old man with a lipoma of the shoulder, requiring an operative time of 34 minutes. B: A 26-year-old man with a lipoma of the back, requiring an operative time of 56 minutes. C: A 61-year-old man with a lipoma of the upper arm, requiring an operative time of 16 minutes. D: A 62-year-old man with a lipoma of the proximal thigh, requiring an operative time of 27 minutes. Retaining ligaments of low signal intensity are seen running perpendicular to the body surface, extending from the periphery of the tumor (bottom images). The number of retaining ligaments is higher in lesions involving the back (B, bottom image) and lower for lesions involving the upper arm (C, bottom image) and the thigh (D, bottom).

Broken down by operative-time group, 68% of lesions (17 of 25) were in the short group, 16% (4 of 25) were in the middle-group, and 16% (4 of 25) were in the long group. Eleven of the fifteen shoulder lesions (73%) were in the short group, requiring an operative time of 30 minutes or less, and one was in the long group, with an operative time of around 60 minutes. This lesion was one of the seven posterior shoulder lesions. Five of the six extremity lesions (83%) were in the short group, and the sixth was in the medium group, requiring less than 50 minutes. One of the two chest wall lesions was in the short group, and the other chest wall lesion and both torso lesions involving the back were in the long group. The operative time for lesions of the torso was more likely to be long than for lesions of the shoulder (p<0.01) or extremity (p<0.05); these differences were statistically significant. Figure 2 (Fig. 2) shows representative patients. 

## Discussion

Use of the one-inch method can achieve lipoma resection through a small incision by detaching the subcutaneous tumor from its retaining ligaments [5]. The operative time depends on the difficulty of tumor removal, especially detachment of the lesion from its retaining ligaments. We found that operative time is associated with tumor location, with lipomas of the torso requiring longer operative times than those at the shoulder or extremity. The number of retaining ligaments affects the difficulty of the one-inch method, and the torso is rich in these ligaments. The fibrous nature of these ligaments within fibromas explains why using either the squeeze technique or liposuction can be difficult for larger lipomas. A previous report on the squeeze technique describes successful resection of lipomas of the forearm, where the retaining ligaments are fewer, but unsuccessful application of the technique for lipomas of the shoulder, where the retaining ligaments are abundant [4]. Liposuction also yields disappointing results when lipomas are fibrous [2], [8]. 

A previous report notes that subcutaneous fibrous structures – which we presume to contain retaining ligaments – are highest in concentration in the lateral and posterior aspects of the body, with density gradually increasing as one moves posteriorly [9]. Indeed, we had two patients with back lipomas, and both were in the long-operative time group. Of our two patients with chest lipomas, one was in the short group and the other was in the long group. Of our seven patients with shoulder lipomas, one was in the long group: this patient had a posterior shoulder lipoma, which may be anatomically similar to a back lipoma. Therefore, the difficulty of the one-inch method is related not only to the lipoma’s anatomic location on the torso, but also to the lipoma’s posterior position. Lipomas on the back are likely less to be amenable to the one-inch method, and surgeons should be aware that the technique should be chosen carefully for lipomas of the posterior shoulder, as these may require a longer operative time.

Taking into consideration that greater numbers of retaining ligaments are associated with procedure difficulty, it would be useful to conduct a preoperative assessment to determine the concentration of retaining ligaments at the lipoma site. The retaining ligaments are membranous, depicted as linear structures on MRI, and are especially visible on the plane horizontal to the body surface. These ligaments are observed at the periphery of the lipoma, intrude into the tumor, and have low signal intensity on T1- and T2-weighted MRI [5]. 

General anesthesia is recommended for all patients undergoing the one-inch method. We feel that local anesthesia may be an option for procedures that are anticipated to last less than 30 minutes, which would include lipomas of the extremity or the anterior shoulder. However, we recommend preoperative confirmation of a low prevalence of retaining ligaments at the operative site before proceeding with local anesthesia. 

We found that the one-inch method can be used for any large lipoma, even those larger than 10 cm, because of skin laxity [5]. The operative time using the one-inch method is not related to lipoma size; therefore, patients with larger lipomas can benefit from the smaller incision. Unfortunately, the method is actually less ideal for smaller lipomas (<1 inch) because the method will still leave a one-inch scar, which is larger than the removed lipoma. 

During the one-inch method, it may be useful to use Pean forceps to break up the lipoma at the incision site at the beginning of the procedure, or to cut the retaining ligaments with scissors and extract the lipoma in piecemeal fashion. Making the incision at the center of the lipoma is a reasonable way of facilitating detachment of the lipoma from the retaining ligaments. Making the incision at the periphery of the lipoma is possible, but it makes the one-inch method more difficult. 

## Conclusion

Operative time for lipoma removal is dependent on the difficulty of detaching the tumors from their retaining ligaments. The one-inch method is indicated for any large lipoma, although the operative time for lipomas of the torso is longer than for those of the shoulder or extremity. Because the number of retaining ligaments is higher in the back, lipomas of the back could be less amenable to the one-inch method, and surgeons should use caution in selecting a surgical technique for lipomas of the posterior shoulder because they may take longer than other lesions. In contrast, the one-inch method can easily be used for lipomas of the anterior shoulder or the extremity. Preoperative MRI may be able to predict the difficulty of surgery by visualizing the retaining ligaments in an orientation horizontal to the body surface.

## Notes

### Ethics statement

The study was approved by the institutional ethics committee. All patients represented in this study were informed that the data from their case would be de-identified and used in a journal publication.

### Competing interests

The authors declare that they have no competing interests.

## Figures and Tables

**Table 1:**
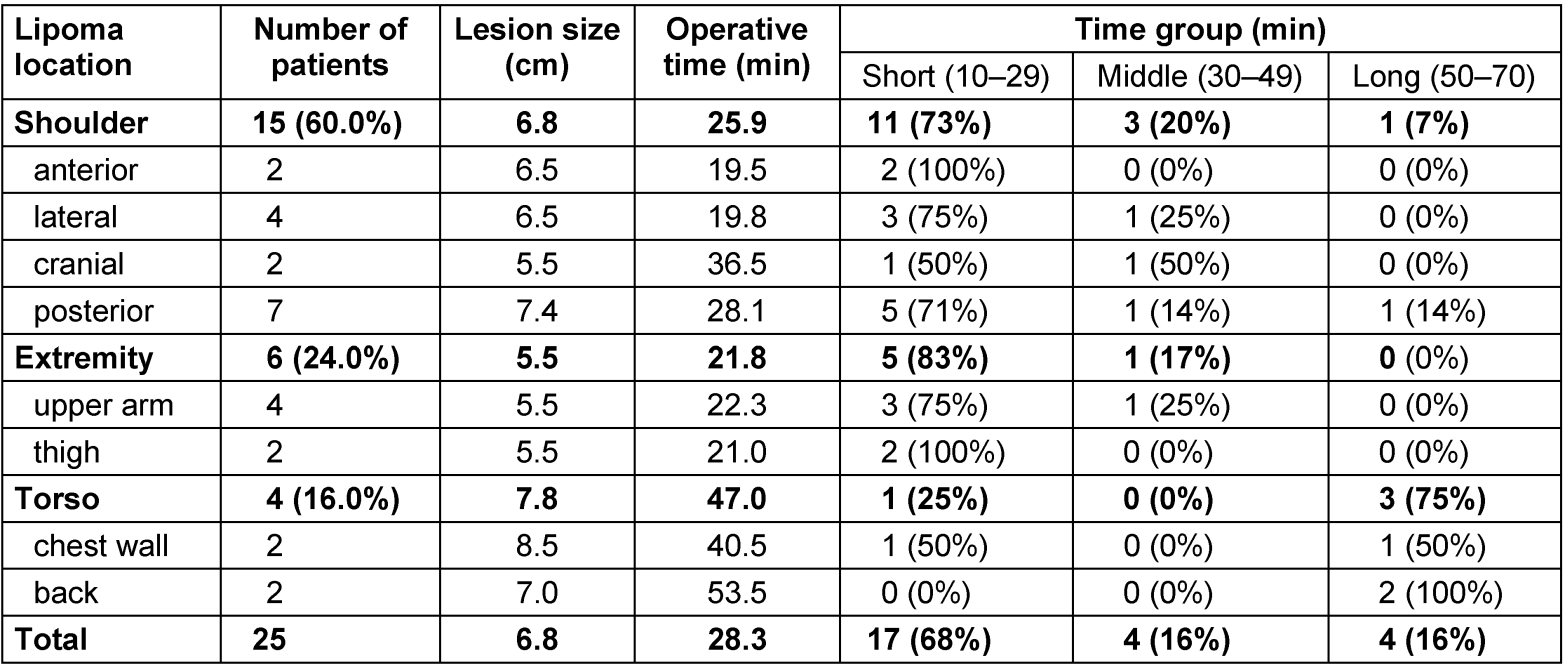
Subcutaneous lipomas treated with the one-inch method
